# Nuciferine Effectively Protects Mice against Acetaminophen-Induced Liver Injury

**DOI:** 10.3390/antiox12040949

**Published:** 2023-04-18

**Authors:** Zixiong Zhou, Jing Qi, Yajiao Wu, Chutao Li, Wenqiang Bao, Xiaohuang Lin, An Zhu

**Affiliations:** 1 Department of Pathology and Institute of Oncology, School of Basic Medical Sciences, Fujian Medical University, Fuzhou 350122, China; zzxpathology@fjmu.edu.cn; 2 Department of Biochemistry and Molecular Biology, School of Basic Medical Sciences, Fujian Medical University, Fuzhou 350122, China; qijing2020@fjmu.edu.cn; 3 Key Laboratory of Ministry of Education for Gastrointestinal Cancer, School of Basic Medical Sciences, Fujian Medical University, Fuzhou 350122, China

**Keywords:** acetaminophen, acute liver injury, nuciferine, oxidative stress, autophagy, anti-inflammatory responses

## Abstract

Acetaminophen (APAP) overdose still poses a major clinical challenge and is a leading cause of acute liver injury (ALI). N-acetylcysteine (NAC) is the only approved antidote to treat APAP toxicity while NAC therapy can trigger side effects including severe vomiting and even shock. Thus, new insights in developing novel therapeutic drugs may pave the way for better treatment of APAP poisoning. Previous research has reported that nuciferine (Nuci) possesses anti-inflammatory and antioxidant properties. Therefore, the objective of this study was proposed to investigate the hepatoprotective effects of Nuci and explore its underlying mechanisms. Mice were intraperitoneally (i.p.) administered with APAP (300 mg/kg) and subsequently injected with Nuci (25, 50, and 100 mg/kg, i.p.) at 30 min after APAP overdose. Then, all mice were sacrificed at 12 h after APAP challenge for further analysis. Nuci-treated mice did not show any side effects and our results revealed that treating Nuci significantly attenuated APAP-induced ALI, as confirmed by histopathological examinations, biochemical analysis, and diminished hepatic oxidative stress and inflammation. The in silico prediction and mRNA-sequencing analysis were performed to explore the underlying mechanisms of Nuci. GO and KEGG enrichment of the predicted target proteins of Nuci includes reactive oxygen species, drug metabolism of cytochrome P450 (CYP450) enzymes, and autophagy. Furthermore, the mRNA-sequencing analyses indicated that Nuci can regulate glutathione metabolic processes and anti-inflammatory responses. Consistently, we found that Nuci increased the hepatic glutathione restoration but decreased APAP protein adducts in damaged livers. Western blot analysis further confirmed that Nuci effectively promoted hepatic autophagy in APAP-treated mice. However, Nuci could not affect the expression levels of the main CYP450 enzymes (CYP1A2, CYP2E1, and CYP3A11). These results demonstrated that Nuci may be a potential therapeutic drug for APAP-induced ALI via amelioration of the inflammatory response and oxidative stress, regulation of APAP metabolism, and activation of autophagy.

## 1. Introduction

Nuciferine (Nuci), an aporphine alkaloid isolated from *Nelumbo nucifera* Gaertn, has been widely used in both daily diet and traditional Chinese medicine [1]. It has been clinically used for hemorrhage and postpartum blood halo according to the Chinese Pharmacopoeia and also reported with the properties of anti-obesity, anti-oxidation, anti-hyperglycemia, anti-bacteria, anti-tumor, and anti-cerebral ischemia in experimental models [2]. To the organs’ protection effect, Nuci exerts excellent pharmacological therapy in disease models of kidney, liver, bone, and nervous system [3]. In the research area of liver injury, most reports focus on the Nuci-mediated metabolism regulation through glutathione (GSH), superoxide dismutase, inflammatory cytokines, and bile acid, so the liver metabolic disease models such as non-alcoholic fatty liver disease and hepatocellular steatosis are widely applied for investigation [4]. However, the liver metabolic disease of rodents needs a long time to construct models, which belong to non-acute exposure. Whether the Nuci still exerts amelioration effects in the acute liver injury (ALI) model is still unknown.

Acetaminophen (APAP) is the major metabolite of phenacetin and is commonly used as a clinical antipyretic analgesic, even recommended by the World Health Organization as the preferred antipyretic drug for infants and children over 2 months of age with high fever [5]. APAP is rapidly absorbed from the gastrointestinal tract after oral administration, then evenly distributed in the body, and the blood concentration reaches a peak within 0.5–2 h [6]. Most of the APAP in the human body is combined with glucuronic acid and sulfuric acid to form a non-toxic substrate that is excreted from urine, and a small amount of the drug is excreted from the urine in its native form [7]. About 5% of APAP in the liver is dependent on the metabolism of cytochrome P450 (CYP450) enzyme to produce a highly active intermediate metabolite, N-acetyl-p-benzoquinone imine (NAPQI). NAPQI can be stably covalently bonded to glutathione (GSH) in the central lobular cells of the liver and lose its toxicity, and it is then excreted by the kidney [8]. APAP is well tolerated at a therapeutic dose with few adverse effects and is available over the counter in most countries [9]. However, in the case of overdose APAP, glycolaldehyde and sulfation pathways are saturated, and a large amount of NAPQI is accumulated and rapidly covalently bound to GSH, leading to glutathione depletion. As a result, excessive NAPQI will combine with the macromolecular protein mercapto of hepatocytes and induce necrosis [10]. As a drug with an acetanilide structure, its medicinal safety attracts a great deal of attention. The Food and Drug Administration of the USA suggested that drugs containing more than 325 mg of APAP in a single dose should not be prescribed or dispensed, since an overdose of APAP can lead to ALI and even death [11].

APAP has been included in the pharmacopeias of China, America, Japan, and Britain, but the adverse reactions and severe liver toxicity caused by the abuse and overdose of the drug have attracted more and more attention [12]. Sufficient evidence from systematic reviews, clinical epidemiological data, case reports, as well as in vitro and in vivo experiments have demonstrated the liver side effects of APAP [13,14]. It is an urgent need to prevent hepatotoxicity and promote the rational and safe use of APAP. In the present study, we will conduct an ALI model induced by APAP in C57BL/6 mice, then use Nuci to assess its ameliorative effect on APAP-induced ALI. The next generation of high-throughput mRNA sequencing will be performed to analyze the liver injury induced by APAP and the therapeutic effect of Nuci. The biomarkers of oxidative stress, inflammatory response, CYP450 metabolic enzymes, and autophagy are detected to reveal the underlying molecular mechanisms.

## 2. Materials and Methods

### 2.1. Preparation of Nuci

**Figure 1:**
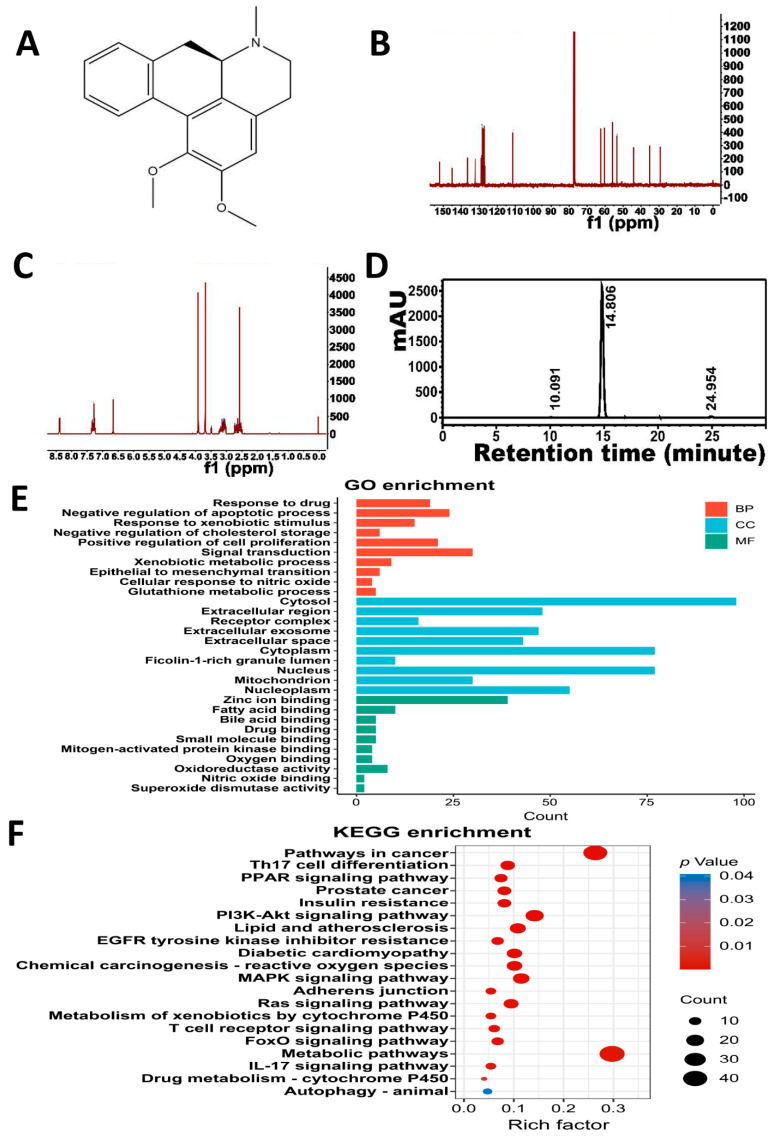
GO and KEGG enrichment of the predicted target proteins of Nuci. (**A**) The chemical structure of Nuci. The (**B**) 400 MHz ^13^C and (**C**) ^1^H NMR spectra of Nuci. (**D**) The purity of Nuci was determined by HPLC. Chromatographic conditions: Sinachrom ODS BP (250 × 4.6 mm, 5 μm particle size); mobile phase of methanol: 1% triethylamine = 75:25; column temperature: 40 °C; injection volume: 20 μL. The retention time of Nuci was 14.806 min, and the area percent of Nuci was 98.918%. The (**E**) GO and (**F**) KEGG enrichment analysis of potential target proteins of nuciferine.

Nuci (CAS#475-83-2) was purchased from MUST Bio-Technology Co., Ltd. (Chengdu, China). The chemical structure of Nuci (Figure 1A) was confirmed by nuclear magnetic resonance (NMR) of Bruker Avance 400 (Rheinstetten, Germany) (Figure 1B,C). Nuci has a purity of 98.918% (Figure 1D), as quantitatively analyzed by high-performance liquid chromatography (HPLC) of Shimadzu LC20A (Tokyo, Japan) analysis.

### 2.2. Animal Experiments

Eight-week-old C57BL/6 male mice (SPF grade) were bought from Wushi Laboratory Animal (Fuzhou, China). Mice were kept in the following conditions: pathogen-free, 22 °C, 50% relative humidity, and 12 h alternating light/dark cycle. All care and animal experiments in the study were reviewed and approved by the Experimental Animal Ethical Committee of Fujian Medical University (IACUC FJMU 2022-NSFC-0270).

All mice fasted for 16 h before APAP administration. To evaluate the effects of Nuci in APAP-triggered ALI, the mice were i.p. injected with APAP (300 mg/kg), which dissolved in PBS to induce ALI, and were treated at 30 min after APAP overdose with Nuci (25, 50, and 100 mg/kg) by i.p. administration. The non-toxic doses of Nuci were determined based on the previous study [15]. The mice in the control group received the same amount of PBS. At 12 h after APAP administration, mice were sacrificed under anesthesia, and blood samples and liver tissues were collected for further analysis. 

### 2.3. Liver Histopathological Examinations

Paraffin-embedded liver tissues were sectioned at 5 μm. After being dewaxed 3 times with xylene, liver sections were dehydrated by ethyl alcohol, suffered with hematoxylin and eosin staining (H&E staining), and labeled for terminal deoxynucleotidyl transferase-mediated dUTP nick-end labeling (TUNEL) -positive hepatocytes according to the standard instructions. 

To measure the hepatic content of APAP protein adducts, hepatic sections were incubated with anti-APAP polyclonal antibody (Bio-rad, Hercules, CA, USA) at 4 °C overnight. Then, the slides were incubated with a biotinylated secondary antibody (Life-iLab, Shanghai, China) and underwent 3,3′-diaminobenzidine staining and hematoxylin counterstaining according to the manufacturer’s instructions. All liver section images were taken by a light microscope (BX-51, Olympus Corp., Tokyo, Japan).

### 2.4. Biochemical Analyses

For serum levels of liver damage-related enzymes such as alanine/aspartate aminotransferase (ALT/AST) (Nanjing Bioengineering Institute, Nanjing, China) activities, the absorbance at 490 nm was detected with an Emax Precision Microplate Reader (Molecular Devices, San Jose, CA, USA). Hepatic Malondialdehyde (MDA) or glutathione (GSH) levels were measured according to the manufacturer’s instructions (Nanjing Bioengineering Institute, Nanjing, China). MDA can react with thiobarbituric acid to form a red product with a maximum absorption peak at 532 nm. The homogenates of liver tissues and standards were incubated with working solution in a 100 °C water bath for 40 min, cooled with flowing water, and centrifuged (1000× *g*, 10 min). Then, after transferring 200 μL supernatants and standards to a 96-well culture plate, the absorbance was detected at 532 nm. Finally, the hepatic concentration of MDA was calculated according to the standard curve.

### 2.5. Western Blot

Total proteins were extracted using RIPA buffer (Beyotime, Shanghai, China). The protein samples were loaded on SDS-PAGE gel, separated by electrophoresis, and then transferred on a PVDF membrane (Millipore) that was further incubated with primary antibodies for LC-3B, p62, and GAPDH (Cell Signaling Technology, Danvers, MA, USA) after blocking with 5% BSA (Bio-Rad). Next, the bands were visualized by a gel imager with enhanced ECL reagents (Advansta, San Jose, CA, USA), and images were obtained by Electrophoresis Image Analysis System (Peiqing Science & Technology, Shanghai, China). The protein gray densities of internal control and target were quantified by Image J software. 

### 2.6. Quantitative Real-Time Polymerase Chain Reaction (qRT-PCR) 

As described before [16], Trizol reagent (GLPBIO, Montclair, CA, USA) was used to isolate the total RNA of liver samples, which was reverse-transcribed into cDNA by the Hifair^®^ III 1st Strand cDNA Synthesis SuperMix for qRT-PCR (gDNA digester plus) according to the instruction (Yeasen, Shanghai, China). The cDNA obtained in the experiment was perfromed by HRbio™ qPCR SYBR Green Master Mix (Fujian Herui Biotechnology, Fuzhou, China). The primer sequences used were listed in Table 1.

### 2.7. Prediction of Potential Target Proteins of Nuci

The potential target proteins of Nuci were predicted by the PharmMapper server (http://www.lilab-ecust.cn/pharmmapper/, accessed on 10 February 2022) [17]. Firstly, the energy-minimized compound was uploaded to PharmMapper in mol2 format, and the mapping database contains 2241 target proteins. Then, the names of mapped proteins were standardized in the UniProt database (https://www.uniprot.org/, accessed on 20 February 2022). Gene Ontology (GO) and Kyoto Encyclopedia of Genes and Genomes (KEGG) enrichment analyses were performed in the DAVID database (Frederick, MD, USA) [18], and bar plots and bubble plots were drawn using the Hiplot Pro online tool (https://hiplot.com.cn/home/index.html, accessed on 25 February 2022).

### 2.8. mRNA-seq and Bioinformatics Analysis

Total RNA was extracted from three samples per group. The mRNA sequencing library was prepared, and high-throughput sequencing was performed in DNBSEQ-T7 (MGI, Shenzhen, China) of Seqhealth (Wuhan, China). The quality of the sequencing data was evaluated using FastQC, then high-quality reads were aligned to the mouse Ensembl genome using HISAT2 [19]. DESeq [20] was employed to determine the differentially expressed genes (DEGs), which were annotated by GO [21] and KEGG [22]. The gene set enrichment analysis (GSEA) and plot of the heatmap [23] were performed by R software (version 4.2.3). In addition, a heatmap package in R software was performed to parts of DEGs.

### 2.9. Molecular Docking

Molecular docking was performed using SYBYL-X 2.0 software (Tripos, St. Louis, MO, USA) to probe the interactions between the Nuci and autophagy-associated proteins. We obtained 3D structures of proteins from the Protein Data Bank and pre-treated their structures in SYBYL-X 2.0 to add hydrogen atoms, repair side chains, and remove heteroatoms and water molecules [24]. We then downloaded the 2D structure of Nuci from the PubChem database (https://pubchem.ncbi.nlm.nih.gov/, accessed on 5 October 2022) and imported it into Chem3D software (Waltham, MA, USA) to convert it into a 3D structure, according to the principle of energy minimization [25]. The semi-flexible docking method in Surflex-Dock GeomX mode was used in the docking between proteins and Nuci [26]. The total score of docking is a comprehensive factor to evaluate polar complementarity, hydrophobic complementarity, entropy, and solvation. When its value was higher than 5, the docking was considered a stable interaction [27].

### 2.10. Enzyme-Linked Immunosorbent Assay (ELISA) 

To assess the hepatic inflammation, we measured the protein level of interleukin 17 (IL-17) with liver tissue lysates by using a ELISA kit (Invitrogen, Carlsbad, CA, USA) under the direction of the manufacturer.

### 2.11. Statistical Analysis

All experimental data were analyzed by GraphPad 9.0 software and presented as mean ± standard deviation (SD). A non-parametric one-way analysis of variance (ANOVA) was used for multiple-group comparison. The significant difference between groups was marked as *p* < 0.05.

## 3. Result

### 3.1. GO and KEGG Enrichment of the Predicted Target Proteins of Nuci

A total of 164 potential target proteins of Nuci were predicted in the PharmMapper server (Chinese Academy of Sciences, Shanghai, China), and GO and KEGG enrichment of these proteins was performed using the DAVID database (https://david.ncifcrf.gov/, accessed on 23 February 2022), yielding 506 GO entries and 100 KEGG entries with a statistical *p*-value less than 0.05. There were 316 biological processes (BP), 47 cellular components (CC), and 143 molecular functions (MF) in the GO entries, and they mainly included the response to a drug, glutathione metabolic process, bile acid binding, oxidoreductase activity, and superoxide dismutase activity (Figure 1E). The KEGG enrichment terms included reactive oxygen species, IL-17 signaling pathway, drug metabolism of CYP450 enzymes, and autophagy.

### 3.2. Treatment with Nuci Mitigates APAP-Evoked ALI in Mice

**Figure 2:**
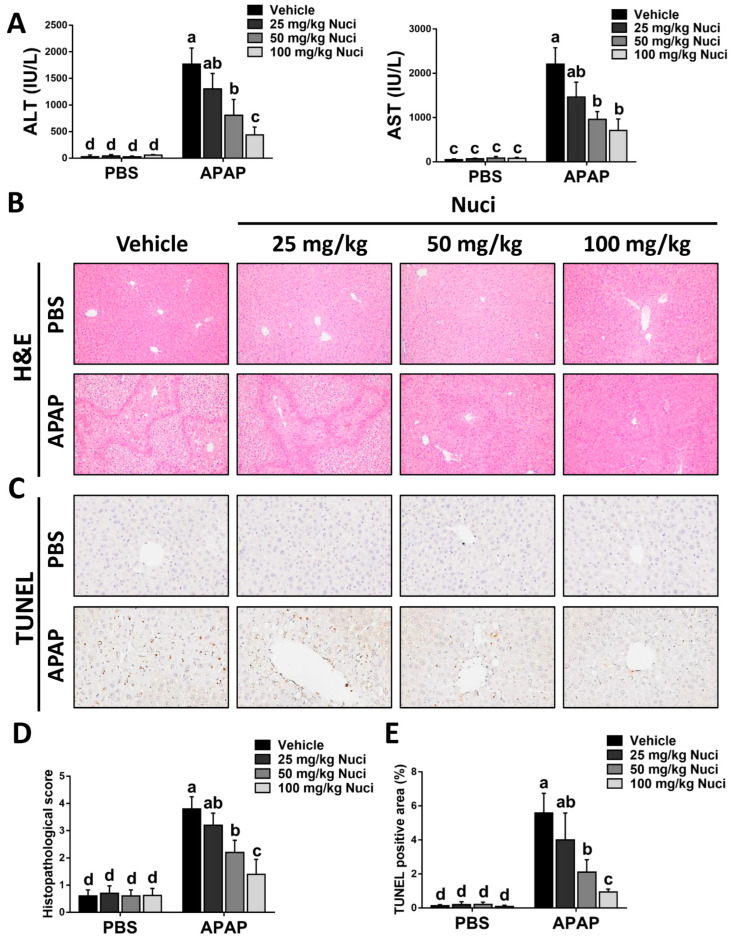
Treatment with Nuci mitigates APAP-evoked ALI in mice. Mice were injected with APAP (300 mg/kg, i.p.) alone or with indicated concentrations of Nuci 30 min after APAP overdose. (**A**) The serum levels of ALT and AST were measured. (**B**–**E**) H&E and TUNEL staining were performed, and the histopathological score and TUNEL-positive cells were assessed. Values are expressed as means ± SD per group. Significant differences between the groups at *p* < 0.05 are represented by different letters. *n* = 8 mice per group. Original magnifications: H&E (200×) and TUNEL (400×).

As shown in Figure 2A–C, severe damage of liver lobules was induced by APAP (300 mg/kg) treatment, as evidenced by serum biochemical and histopathologic analysis. Treating with Nuci showed a dose-dependent amelioration of serum ALT and AST levels in APAP-treated mice (Figure 2A). The histopathologic results also exhibited a similar pattern with the alteration of serum biochemicals (Figure 2B,D). Uniformly, hepatocyte necroapoptosis triggered by an APAP overdose was substantially decreased by Nuci injection, confirmed by TUNEL staining and analysis of TUNEL-positive areas (Figure 2C,E). In line with our findings, several studies also demonstrated that Nuci can ameliorate folic acid-induced nephrotoxicity [28], lipopolysaccharide-evoked pulmonary toxicity [29], and diet-triggered hepatotoxicity [30]. Taken together, these results suggest that treating with Nuci protects mice from APAP-induced ALI.

### 3.3. The Bioinformatic Analysis of mRNA-seq

**Figure 3:**
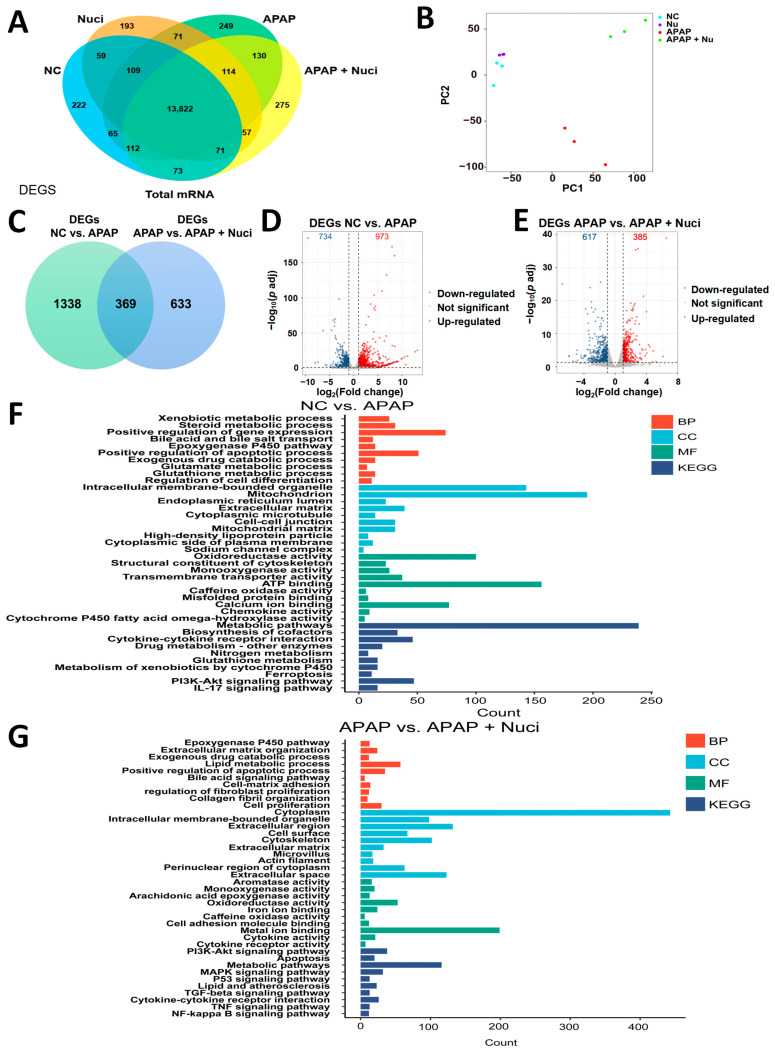
The bioinformatic analysis of mRNA-seq. (**A**) Venn diagram shows the number of mRNAs in NC, Nuci, APAP, and APAP + Nuci groups. (**B**) PCA for the mRNA expression in different groups. (**C**) The number of overlapping genes between NC vs. APAP and APAP vs. APAP + Nuci. Volcano plot of the DEGs in (**D**) NC vs. APAP and (**E**) APAP vs. APAP + Nuci. GO and KEGG pathway enrichment of the DEGs in (**F**) NC vs. APAP and (**G**) APAP vs. APAP + Nuci.

**Figure 4:**
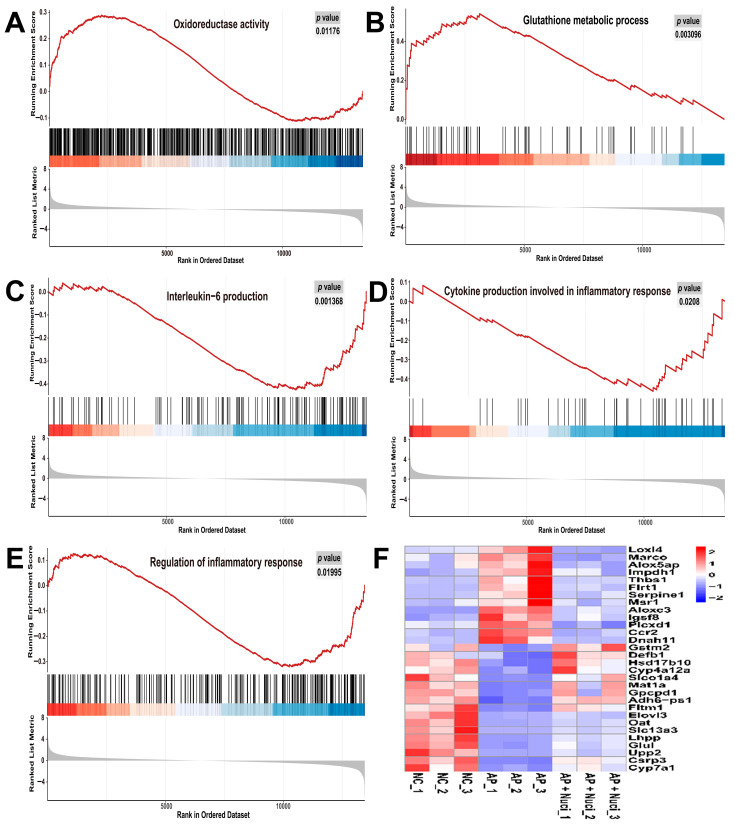
Compared with the APAP-treated group, the GSEA of (**A**) oxidoreductase activity, (**B**) Glutathione metabolic process, (**C**) interleukin-6 production, (**D**) cytokine production involved in the inflammatory response, and (**E**) regulation of inflammatory response genes in APAP + Nuci group. (**F**) Heatmap of parts of DEGs in NC, APAP, and APAP + Nuci groups. *n* = 3.

A total of 13,822 overlapping genes were obtained among NC, Nuci, APAP, and APAP + Nuci groups (Figure 3A). The principal component analysis (PCA) showed a clear dispersion between different groups (Figure 3B). There were 369 overlapped DEGs between NC vs. APAP and APAP vs. APAP + Nuci, based on the DEGs screening criteria of |log2 (Fold Change)| > 1 and *p* adj < 0.05 (Figure 3C). The volcano plots showed that 734 DEGs were down-regulated, while 973 DEGs were up-regulated in NC vs. APAP (Figure 3D). Additionally, 617 DEGs were down-regulated, while 385 DEGs were up-regulated in APAP vs. APAP + Nuci (Figure 3E). GO, KEGG, and GSEA analyses were performed based on the 369 overlapped DEGs. In the NC vs. APAP group, GO terms mainly mapped to steroid metabolic process, epoxygenase P450 pathway, and glutathione metabolic process, while KEGG terms were mapped to drug metabolism, glutathione metabolism, metabolism of xenobiotics by CYP450 enzymes, and IL-17 signaling pathway (Figure 3F). In APAP vs. APAP + Nuci group, GO terms were mainly mapped to the epoxygenase P450 pathway, bile acid signaling pathway, and oxidoreductase activity, meanwhile KEGG terms were mainly mapped to metabolic pathways and TNF signaling pathway (Figure 3G). Compared with the APAP group, the GSEA of APAP + Nuci showed that the oxidoreductase activity and glutathione metabolic process were activated, while the interleukin-6 production, cytokine production involved in an inflammatory response, and regulation of inflammatory response were inhibited (Figure 4A–E). The heatmap of vital DEGs was plotted in Figure 4F, and the therapeutic effect of Nuci in APAP-induced mRNA abnormal expression was obvious.

### 3.4. Treating Nuci Decreases the Formation of APAP Protein Adducts in Damaged Murine Livers

**Figure 5:**
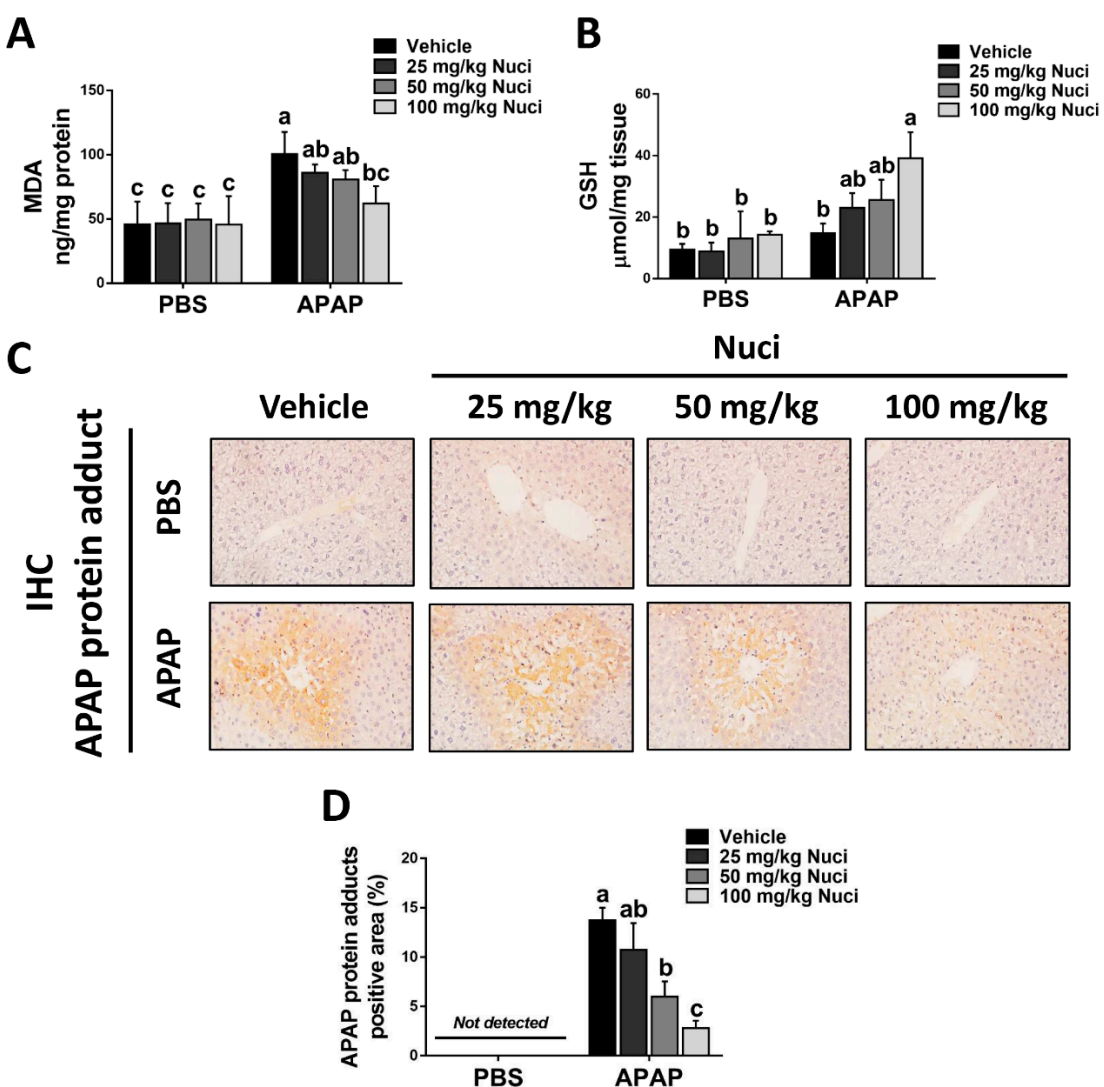
Treating Nuci decreases the formation of APAP protein adducts in damaged murine livers. Mice were administered with APAP (300 mg/kg) and subsequently injected with indicated doses of Nuci at 30 min after APAP overdose. Then, liver samples were collected at 12 h after APAP challenge. The hepatic levels of MDA (**A**) and GSH (**B**) were measured. (**C**,**D**) The hepatic content of APAP protein adducts was checked by IHC staining, and the positive area was qualified. Values are expressed as means ± SD per group. Significant differences between the groups at *p* < 0.05 are represented by different letters. *n* = 8 mice per group. Original magnifications: IHC (400×).

Given the fact that APAP is metabolized by CYP450 enzymes (isoforms CYP1A2, 2E1, and 3A11) in mouse hepatocytes [31], we first evaluated whether Nuci treatment altered the expression levels of these CYP450 isoforms. Our data revealed that Nuci injection did not alter the mRNA levels of these CYP450 isotypes (Figure S1). The toxic metabolite of APAP can deplete hepatic GSH and modify cellular proteins. The data show that treated-Nuci decreased MDA levels in injured livers and parallelly statistically up-regulated hepatic GSH levels in a dose-dependent manner in the mice with APAP overdose (Figure 5A,B). Since Nuci treatment promotes hepatic GSH synthesis, we assumed that Nuci may affect the APAP metabolism. As shown in Figure 5C,D, the formation of APAP protein adducts was substantially reduced by Nuci in injured livers in a dose-dependent manner. Altogether, our data indicated that the hepatoprotective effects provided by Nuci may be relevant to its inhibition of APAP metabolism in mice.

### 3.5. Nuci Promotes Hepatic Autophagy after APAP Overdose

**Figure 6:**
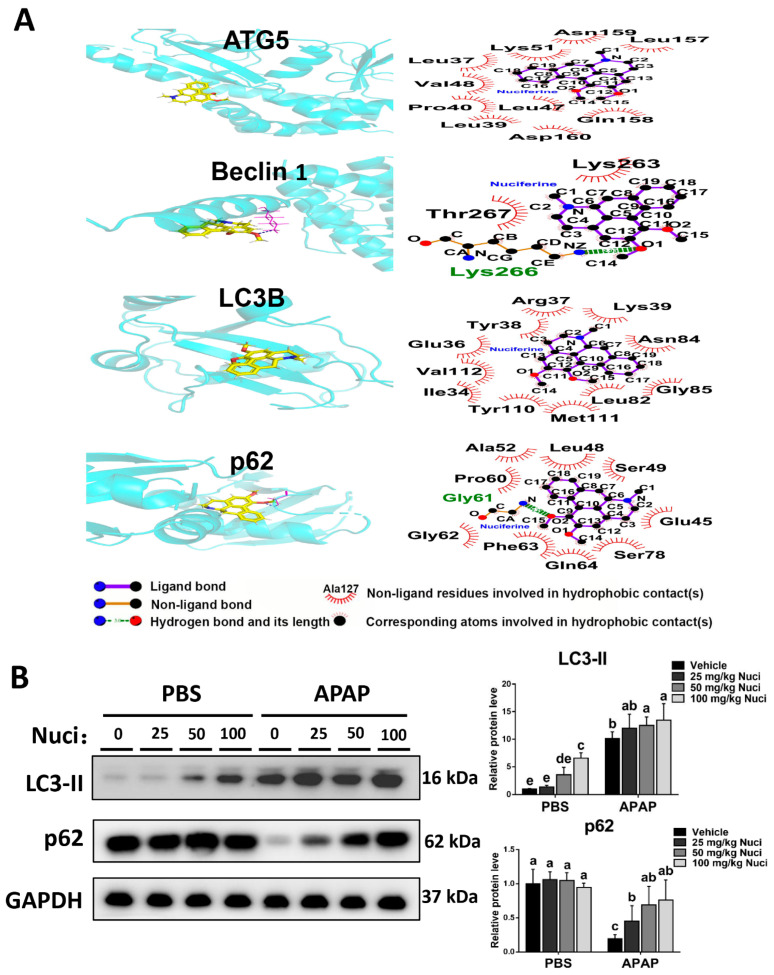
Nuci promotes hepatic autophagy after APAP overdose. (**A**) The interactions between Nuci and autophagy-associated proteins. (**B**) Mice were administered with APAP (300 mg/kg) and subsequently injected with Nuci (25, 50, and 100 mg/kg) at 30 min after APAP overdose. Then, liver samples were collected at 12 h after APAP challenge. Liver extracts were immunoblotted with anti-LC3-Ⅱ, p62, and GAPDH antibodies, and the blot density was qualified by ImageJ software. Each lane represents one individual animal (*n* = 3 mice per group). Values are expressed as means ± SD per group. Significant differences between the groups between groups at *p* < 0.05 are represented by different letters.

Accumulating evidence to date has revealed that sustained autophagy could relieve APAP-induced liver injury by modulating oxidative stress [32]. The interactions between Nuci and autophagy proteins [Beclin 1, autophagy protein (Atg) 5, microtubule-associated protein 1 light chain 3 (LC3)-Ⅱ, and p62] were predicted by molecular docking, and the 2D and 3D interaction diagrams were shown in Figure 6A. Theoretically, Nuci bound to autophagy proteins via hydrophobic contacts and hydrogen bonds (Table 2). All the total scores for Beclin 1, LC3-Ⅱ, and p62 were higher than 5, indicating stable interactions between Nuci and autophagy proteins. Consistently, Western blot analysis illustrated that the protein levels of LC3-II and p62 were dose-dependently increased by supplementing with Nuci in injured livers (Figure 6B). Therefore, these responses indicated that Nuci-induced autophagy may contribute to the amelioration effects of APAP-induced ALI.

### 3.6. Nuci Inhibits APAP-Induced Sterile Inflammation Responses in Mice with ALI

**Figure 7:**
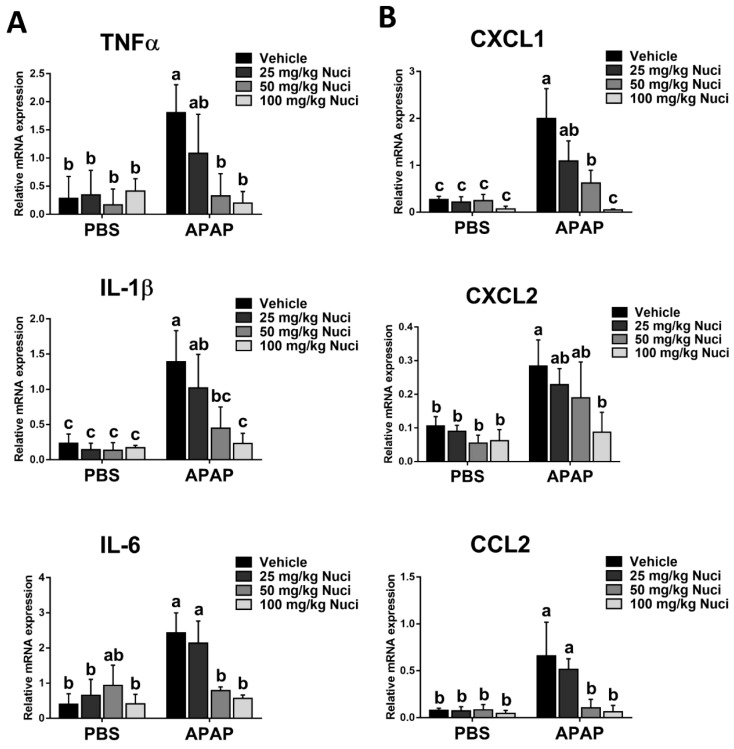
Nuci inhibits APAP-induced sterile inflammation responses in mice with ALI. Mice were administered with APAP (300 mg/kg) and subsequently injected with indicated doses of Nuci at 30 min after APAP overdose. Then, liver samples were collected at 12 h after APAP challenge. Gene expression was measured by qRT-PCR and normalized to GAPDH expression. (**A**) The mRNA expression levels of inflammatory cytokines including TNF-α, IL-1β, and IL-6 were detected. (**B**) The mRNA expression levels of CXCL1, CXCL2, and CCL2 were measured. Values are expressed as means ± SD per group. Significant differences between the groups at *p* < 0.05 are represented by different letters. *n* = 8 repeats per group.

Since the infiltration of immune cells and its evoked hepatic inflammatory responses play a key role in the pathophysiology of APAP-induced ALI, we next examined whether treating Nuci affects APAP-induced hepatic inflammation. As shown in Figure 7A, APAP remarkably increased the mRNA expression levels of TNF-a, IL-1β, and IL-6 compared to the control group, whereas Nuci treatment diminished the production of inflammatory cytokines induced by APAP administration. Additionally, Nuci treatment markedly reduced the mRNA levels of C-X-C motif ligand (CXCL) 1, CXCL2, and C–C motif chemokine ligand 2 (CCL2) (Figure 7B). However, treating-Nuci did not significantly alter the production of IL-17 in both PBS-treated and APAP-treated livers (Figure S2). These findings indicated that Nuci suppresses hepatic inflammation, which decreased APAP-induced ALI.

## 4. Discussion

During the inflammatory response, arachidonic acid in cells synthesizes prostaglandins and other inflammatory cytokines through cyclooxygenase and leads to the symptoms of tissue redness, swelling, and heat [33]. APAP acts to regulate body temperature and relieve pain by inhibiting the enzyme cyclooxygenase, which is required for the synthesis of prostaglandins [34]. In China, APAP is also widely recommended for adults and children with fever and pain caused by colds and headaches. It is the most consumed over-the-counter analgesic in the United States. Among the medicinal overdose reported to the poison control centers of America, APAP overdose is the most common one [35]. Excessive or long-term use of APAP can cause side effects including liver damage, cholestatic hepatitis, and, in severe cases, hepatic coma and death [36]. Population-based surveillance for acute liver failure (ALF) reported that APAP poisoning is the most common etiology [37]. In the United States, nearly 50% of adult cases of acute liver dysfunction are caused by drugs, among which APAP is the first drug causing liver failure [38]. In September 2017, after an assessment by the Pharmacovigilance Risk Assessment Committee of the European Medicines Agency, the marketing suspension of extended-release APAP formulations was recommended mainly because of the complex pattern of in vivo release and raised concerns about the safety of extended-release APAP [39]. In the present study, C57BL/6 mice models of ALI were established by single intraperitoneal injection of 25, 50, and 100 mg/kg APAP. After the biochemical detection of sera, the liver enzyme activities of ALT and AST were significantly increased in the APAP-treated groups, compared to the PBS control group. The H&E histopathologic examination observed the destroyed structure of hepatic lobules, the chaotic arrangement of the hepatic cell cord, and the infiltration of inflammatory cells. The IHC staining observed increased APAP protein adducts. The above results indicated established ALI model in the present study.

Traditional drug Nuci is applied to alleviate the APAP-induced hepatotoxicity since it exerts a therapeutic effect through the promotion of GSH production [28], the same antioxidant enzyme for reducing the toxicity of NAPQI. An integrated pharmacophore matching platform was used to predict the protein targets of APAP, and 164 candidate proteins were screened out. The GO and KEGG enrichment terms include response to the drug, glutathione metabolic process, bile acid binding, oxygen binding, oxidoreductase activity, superoxide dismutase activity, and drug metabolism by CYP450. Sequentially, mRNA sequencing was performed and reported 369 genes involved in the Nuci ameliorated APAP-induced ALI. These genes were enriched in the terms of drug metabolism, oxidoreductase activity, glutamate metabolic process, and so on, thus confirming the enrichment terms of Pharmapper prediction. In the present study, the APAP produced excessive NAPQI, and GSH depletion was the main toxicological mechanism of liver injury. After the supplement of Nuci, the promoted production of GSH catalyzed the conversion of NAPQI to the non-toxic cysteine conjugates and mercapturic acid. In the liver enzyme activity, oxidative stress biomarkers, H&E examination, TUNEL, and IHC staining, the protective effects of Nuci against APAP-induced liver injury were obvious.

It has been demonstrated that APAP triggers autophagy via the activation of epidermal growth factor receptor, transcription factor EB, PINK1/Parkin, and classical phosphatidylinositol 3-kinase/protein kinase B/mammalian target of rapamycin (PI3K/AKT/mTOR) pathway. As a self-digestion process mediated by lysosomes, autophagy inhibits tissue damage by removing misfolded proteins, damaged organelles, and reactive oxygen species [40]. It includes initiation, nucleation, fusion, and hydrolysis between autophagosome and lysosome and is mainly performed by autophagy-related proteins (Atg). Once encountering stress conditions such as nutrient deficiency and hypoxia, activated autophagy activating protein 1 (ULK1) regulates the recruitment of vacuolar sorting protein 34 (Vps34) complex containing autophagy-related protein 14 antibodies (Atg14L). In turn, ULK1 phosphorylates the autophagy effector Beclin-1, thereby enhancing the activity of the Vps34 complex [41]. The autophagy–lysosome system is a classical protein degradation pathway. The autophagosome precursors undergo expansion, elongation, and nucleation and are sequestered into double-layered spherical autophagosomes [42]. At the same time, Atg12 and LC3 regulate autophagosome expansion and closure. With the assistance of Atg3 and Atg7 proteins, autophagosomes fuse with lysosomes to form autolysosomes, which then degrade their contents [43]. p62 is a ubiquitin-binding protein that directly interacts with LC3 and induces its specific degradation through autophagy. In the absence of Atg expression or when the fusion of autophagosome and lysosome is blocked, p62 will accumulate and lead to cytotoxicity, oxidative stress, and DNA damage [44]. In the present study, the molecular docking method was applied to predict the interaction between Nuci and autophagy proteins of ATG5, Beclin 1, LC3-II, and p62, thus preliminarily estimating whether Nuci could activate autophagy. Western blot analysis showed that the protein levels of LC3-II and p62 were increased in Nuci-treated groups regardless of APAP injection. Therefore, the increased autophagy by Nuci may contribute to the alleviation of APAP-induced oxidative damage.

Nuci has been reported to exert anti-inflammatory effects in the models of LPS-induced mice mammary gland epithelial cells, the microglial cells of BV2 mice, RAW264.7 macrophages, and uric acid-treated HK-2 cells, as well as in vivo models of mouse mammary adenitis and rat cerebral ischemia [29,45,46,47]. It activated peroxisome proliferator-activated receptors (α and γ) and inhibited Toll-like receptor 4-mediated nuclear factor-κb and NOD-like receptor family pyrin domain containing 3 inflammasome signaling pathway, thus decreasing the secretion of inflammatory cytokines of TNFα, IL-1β, and IL-6 [48]. APAP overdose causes severe hepatocyte necrosis, which subsequently results in the release of damage-associated molecular patterns (DAMPs) into the systemic circulation. These DAMPs then trigger a sterile inflammatory response resulting in increased cytokine/chemokine formation and recruitment of innate immune cells including macrophages and neutrophils into the liver. In the present study, multiple pro-inflammatory cytokines of TNFα, IL-1β, IL-6, CXCL1, CXCL2, and CCL2 were increased after the APAP challenge and reduced to the normal range with the therapy of Nuci at the dose of 100 mg/kg. Therefore, these results demonstrated that the global inflammatory condition was substantially improved in damaged livers after Nuci treatment.

To characterize the pharmacokinetics, tissue distribution, excretion, and bioavailability of Nuci, a liquid chromatography-tandem mass spectrometry method was validated. After intravenous injection or oral administration of Nuci, the absolute bioavailability was as low as 1.9 ± 0.8% in rats. The AUC_0_→_4 h_ in tissues were in descending order of kidney, lung, spleen, liver, brain, and heart [49]. It is still unknown whether the native form of Nuci or the metabolite is responsible for its therapeutic effects of antioxidant and anti-inflammation. In our previous study, a total of 55 metabolites of Nuci were detected in the plasma, bile, urine, and feces of C57 mice, and their structures were identified by high-resolution mass spectrometry-based metabolomics. Multiple CYP450 and UDP-glucuronosyltransferases and sulfotransferases participated in the metabolism of Nuci by in vitro recombinant enzyme screening assays [50]. After oral administration of APAP, 90% of them were metabolized to non-toxic glucuronide and sulfate metabolites, 2% was directly excreted by the urine, and the remaining fraction was mainly metabolized by CYP2E1 enzyme into NAPQI [51]. In this study, the mRNA expression levels of CYP1A2, CYP2E1, and CYP3A11 were detected, and Nuci did not alter the CYP enzymes at the transcriptional level.

## 5. Conclusions

Our finding indicated that APAP can cause ALI in mice, manifested as lipid peroxidation, GSH depletion, hepatocyte necrosis, and tissue inflammatory infiltration. The Nuci from traditional Chinese medicine exerted its protection and amelioration effects on APAP-induced ALI via the antioxidant system, anti-inflammatory response, regulation of APAP metabolism, and autophagy activation, rather than the influence on CYP metabolic enzymes. The in silico prediction and mRNA high-throughput sequencing also provided evidence to support experimental results. The study revealed the underlying pharmacological mechanism of Nuci on APAP-induced ALI.

## Figures and Tables

**Table 1:** Primer sequence of real-time PCR.

Gene	Gene Accession Number	Forward (5′-3′)	Reverse (5′-3′)
*CYP2e1*	NM_021282	AAGCGCTTCGGGCCAG	TAGCCATGCAGGACCACGA
*CYP1a2*	NM_009993.3	GGTCAGAAAGCCGTGGTTG	GACATGGCCTAACGTGCAG
*CYP3a11*	NM_007818.3	CGCCTCTCCTTGCTGTCACA	CTTTGCCTTCTGCCTCAAGT
*TNFα*	NM_013693.3	GTCTACTCCCAGGTTTCTCTTCAAGG	GCAAATCGGCTGACGGTGTG
*IL-1β*	NM_008361.4	CTCGCAGCAGCACATCAACA	CCACGGGAAAGACACAGGTA
*IL-6*	NM_001314054.1	CAACGATGATGCACTTGCAGA	CTCCAGGTAGCTATGGTACTCCAGA
*CXCL1*	NM_008176.3	TGCACCCAAACCGAAGTC	GTCAGAAGCCAGCGTTCACC
*CXCL2*	NM_009140.2	GCCAAGGGTTGACTTCAAGAACA	AGGCTCCTCCTTTCCAGGTCA
*CCL2*	NM_011333.3	AGCAGCAGGTGTCCCAAAGA	GTGCTGAAGACCTTAGGGCAGA
*GAPDH*	NM_001411840.1	ACGGCAAATTCAACGGCACAG	GAAGACTCCACGACATACTCAGCAC

CYP2e1, Cytochrome P450 2E1; CYP1a2, Cytochrome P450 1A2; CYP3a11, Cytochrome P450 3A11; TNFα, tumor necrosis factor; IL-1β, interleukin-1beita; IL-6, interleukin-6; CXCL1, C-X-C motif ligand 1; CXCL2, C-X-C motif ligand 2; CCl2, chemokine (C-C motif) ligand 2; GAPDH, glyceraldehyde-3-phosphate dehydrogenase.

**Table 2:** Molecular interactions between Nuci and autophagy-associated proteins.

Protein	PDB ID	Total Score	Crash	Polar	H-Bond Number	Residues Involved in H-Bond Formation	Hydrophobic Contacts Number	Residues Involved in Hydrophobic Contacts
Beclin 1	7BL1	5.895	−0.558	1.366	1	Lys266	2	Thr267, Lys263
ATG5	7W36	4.832	−2.124	0.004	0	-	10	Leu157, Asn159, Lys51, Leu37, Val48, Pro40, Leu47, Leu39, Asp160, Gln158
LC3-Ⅱ	5MS2	5.588	−1.523	0	0	-	11	Asn84, Lys39, Arg37, Tyr38, Glu36, Val112, Ile34, Tyr110, Met111, Leu82, Gly85
p62	6JM4	6.713	−1.534	0.892	1	Gly61	9	Gly62, Phe63, Gln64, Ser78, Glu45, Ser49, Leu48, Ala52, Pro60

## Data Availability

All data are included in this manuscript.

## Supplementary Materials

The following supporting information can be downloaded at: https://www.mdpi.com/article/10.3390/antiox12040949/s1, Figure S1: Nuci could not affect the expression levels of main CYP450 enzymes in APAP-treated mice; Figure S2: Nuci could not significantly alter the production of IL-17 in damaged livers.
