# Supplementary material for: Nuciferine Effectively Protects Mice against Acetaminophen-Induced Liver Injury
Source: Antioxidants (Basel). 2023 Apr 18;12(4):949. doi: 10.3390/antiox12040949 (PMC10136285; doi:10.3390/antiox12040949)
Supplement: Supplementary file 1 [file antioxidants-12-00949-s001.zip › antioxidants-2311879-supplementary.pdf]

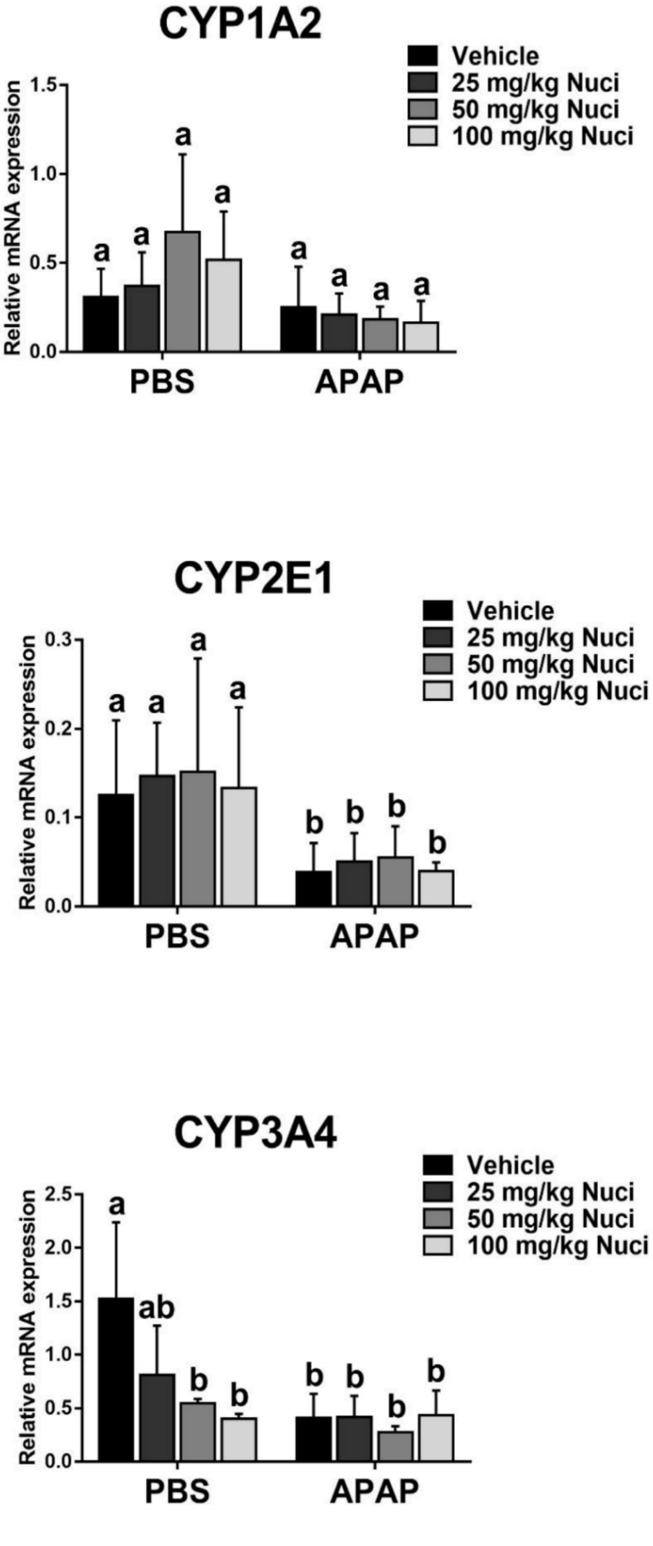

Figure S1. Nuci could not affect the expression levels of main CYP450 enzymes in APAP-treated mice. The expression levels of CYP1A2, CYP2E1, and CYP3A4 were measured. Values are expressed as means ± SD per group (n=8 mice per group).

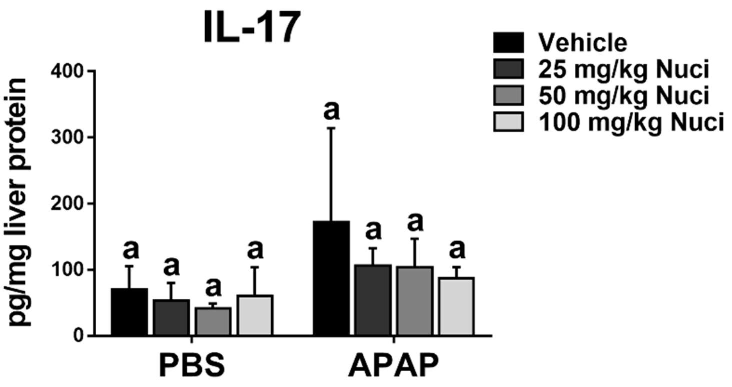

Figure S2. Nuci could not significantly alter the production of IL-17 in damaged livers. The hepatic protein level of IL-17 was measured by using a ELISA kit. Values are expressed as means ± SD per group (n=8 samples per group).
